# Patients’ acceptance of a penicillin allergy de-labelling programme in primary care: a qualitative study

**DOI:** 10.3399/BJGPO.2024.0136

**Published:** 2025-08-27

**Authors:** Marta Santillo, Caity Roleston, Kelsey Armitage, Catherine Porter, Joanne Fielding, Marta Wanat, Shadia Ahmed, Sinisa Savic, Christopher Butler, Sue Pavitt, Jonathan Sandoe, Sarah Tonkin-Crine

**Affiliations:** 1 Primary Care Health Sciences, University of Oxford, Oxford, UK; 2 Faculty of Medicine and Health, University of Leeds, Leeds, UK

**Keywords:** penicillin allergy testing, penicillins, primary health care, qualitative research

## Abstract

**Background:**

About 6% of the UK general practice population has a record of a penicillin allergy but fewer than 10% of these people are likely to be truly allergic. Consequently, a significant portion of the population is denied first-line antibiotics. The ALlergy AntiBiotics And Microbial resistAnce (ALABAMA) trial aimed to determine if a penicillin allergy assessment pathway (PAAP) was safe and effective in de-labelling patients as allergic and improving antibiotic prescribing and patient health outcomes.

**Aim:**

To investigate patients’ experiences of penicillin allergy testing (PAT) and their acceptance of de-labelling following a negative allergy test.

**Design & setting:**

This was a qualitative study using semi-structured interviews with patients who took part in the PAAP intervention arm of the ALABAMA trial.

**Method:**

As part of a mixed-methods process evaluation embedded in the ALABAMA trial, we conducted interviews with patients in the PAAP intervention arm. Data from interviews with patients were analysed using thematic analysis.

**Results:**

Of the 28 participants interviewed, two received a positive PAT result and 26 received a negative PAT result; of these, 24 accepted and two declined de-labelling. At point of trial recruitment, many patients already doubted that they were allergic to penicillin. Patients were happy to attend PAT and felt cared for and safe at the hospital. These factors led to most people trusting their negative test result and accepting de-labelling.

**Conclusion:**

The patients we interviewed engaged with the PAAP intervention and, when testing negative, were predominantly willing to have their allergy record changed and to take penicillin in future. We highlight factors that influenced patients’ acceptance of de-labelling to facilitate future adoption of PAAP. These factors, which should be considered when planning for penicillin allergy testing services, were as follows: patients identifying themselves as low risk before the test; PAT being perceived as trustworthy and safe; patients’ previous experience of penicillin allergy and reactions; patients’ understanding of penicillin reactions; and clear communication after de-labelling.

## How this fits in

Incorrect penicillin allergy records are common and deny people first-line antibiotics. Patients’ views and experiences of penicillin allergy testing (PAT) and de-labelling are poorly understood, as is to encourage acceptance of a change of allergy status. This article contributes to our knowledge by highlighting that it is important to: emphasise that patients are at ’low risk’ of having a true allergy where appropriate; provide a caring environment for PAT; inform patients about the difference between allergic reactions and side effects; and provide consistent information about de-labelling across primary and secondary care settings. This study impacts current management guidelines because it encourages clinicians to discuss and address patient concerns that will help to support de-labelling in primary and secondary care. PAT pathways need to support patients in making them feel safe when undergoing allergy testing, helping them understand the difference between allergy and side effects of medication, and providing consistent messaging across healthcare settings.

## Introduction

When formally tested, fewer than 1 in 10 patients with a penicillin allergy record are found to be truly allergic.^1,2^ The consequences of incorrect penicillin allergy labels include longer hospitals stays^3^ and higher rates of infections with methicillin-resistant *Staphylococcus aureus* and *Clostridioides difficile*.^4^ In addition to this, patients are more likely to be prescribed non-penicillin antibiotics, which are often more expensive^5^ and are associated with higher risk of treatment failure.^6^

The reasons for incorrect penicillin allergy labels are complex. Sometimes side effects of antibiotics include symptoms that are similar to allergic reactions. Symptoms of infections, such as a viral rash, can also be mistaken for an allergic reaction.^7^ Moreover, it can be difficult to confirm a past diagnosis of penicillin allergy as general practice medical records can be incomplete and patients do not recall the ‘allergic’ event.^8,9^

The gold standard to test for penicillin allergy is a drug provocation test (DPT, also known as an oral or intravenous challenge test).^10^ However, UK guidelines advise that patients should undertake a skin prick and/or an intradermal test first,^11^ although a recent update endorses a direct DPT for low-risk patients.^12^ Currently, most patients who are eligible to undergo penicillin allergy testing (PAT) are not offered PAT owing to lack of testing capacity.^13^ Patients who test negative can be ’de-labelled’, meaning their allergy record can be removed. A rapid review on patients’ and clinicians’ views on PAT identified scant literature and no qualitative studies on patients’ experiences of the allergy testing process.^14^ Since then, qualitative work has reported that the factors affecting patients’ willingness to undertake a test and accept de-labelling were the personal relevance and perceived benefits of testing; the importance of safety and perceived risks of testing; and confidence in the test result.^15^ Moreover, interviews highlighted that patients’ views on testing depend on their recollection of their first allergic reaction, and on how they ’*make sense*’ of their allergy status.^16^

The ALlergy AntiBiotics And Microbial resistAnce (ALABAMA) programme aimed to develop a behavioural intervention package for UK general practice to effectively and safely amend incorrect penicillin allergy records. The ALABAMA trial was a multicentre, two parallel-arm, open-label, individually randomised pragmatic trial with embedded process evaluation and cost-effectiveness evaluation.^17^ Participants were randomised to either usual care or to receive the penicillin allergy assessment pathway (PAAP) intervention. PAAP targeted low-risk patients who did not have a history of anaphylaxis or other severe allergic reaction. The PAAP was an efficient one-stop procedure for PAT.

There is limited qualitative research on the experience of patients who have undertaken PAT and had their records changed after a negative test result.^15^ Our study aimed to investigate patients’ experiences of the de-labelling process, as part of the ALABAMA trial, identifying what factors may affect patients in accepting a change of allergy status and consequently taking penicillin following a negative test.

## Method

### Participants and procedure and design

This was a qualitative study using semi-structured interviews with patients who took part in the PAAP intervention arm of the ALABAMA trial. Details about the process, methods, and outcome measures of the interventions have been published elsewhere.^18^ The trial recruited patients assessed as ’low-risk’, which meant they did not have a history of severe allergic reactions such as anaphylaxis. The intervention also aimed to streamline the testing process by screening patients in general practice and by introducing an efficient one-stop PAT at a hospital testing clinic.

### The intervention

Development of the intervention has been described previously.^18^ The PAT included either a skin test followed by an oral challenge test, or direct oral challenge test, depending on the specific patient history. Before attending the test at the hospital clinic, patients in the PAAP arm received a ’Going for a test’ booklet, which provided information on incorrect allergy records, why they would benefit from PAT, and what the test would involve. Following the test, patients received the test result by letter, together with a ’Negative test result’ leaflet (when applicable) and a card confirming the negative result to show other healthcare professionals (HCPs) as needed. The ’Negative test result’ leaflet informed patients of the reliability of the PAT and consequences of a negative test result.

### Recruitment

Patients were invited to take part in an interview by email. Patients were purposively sampled from the intervention arm to include participants with negative test results who accepted de-labelling. We also invited some patients who tested positive and two patients who we were aware had declined de-labelling.

### Interviews

We conducted semi-structured interviews following a topic guide informed by previous literature on penicillin allergy.^14,15^ The topic guide included questions around allergy history, impact of their allergy status on their care, motivations to take part in the trial, experience of being part of the trial, experience of PAT, feelings about the test result, intentions or experiences of taking penicillin when needed in the future (when tested negative). The topic guide remained flexible to participants’ experiences.

We obtained verbal consent from participants at the start of interviews, which was audio-recorded. The interviews were conducted over the telephone by two experienced qualitative researchers, MS and CR (both PhD qualified with substantial previous experience conducting qualitative health research). The researchers introduced themselves, and explained their own expertise and that they were not part of the trial team. The interviews were audio-recorded and then transcribed verbatim by an independent transcriber. Transcripts were then pseudo-anonymised by the researchers.

### Analysis

Data collection and analysis were conducted concurrently. Transcripts of interviews were analysed using inductive thematic analysis.^19^ First, MS familiarised herself with the dataset by reading and re-reading the interview transcripts. Second, MS generated initial codes that captured the important features of the data that addressed the research question on patients’ experiences of PAT. Third, MS grouped the codes into categories. MS created an initial coding manual based on the first six transcripts, which included a definition of the category, which codes were grouped under that category, and examples of quotes. ST-C (PhD qualified, health psychologist, and experienced primary care qualitative researcher) read and coded a sample of the six transcripts. ST-C reviewed the first draft of the coding manual. After 12 interviews were conducted, MS refined the second version of the coding manual. As the interviews were completed, a sample of later transcripts were then read by the wider team, which included MS, CR, and ST-C and a final coding manual was produced, which was applied to the rest of the dataset. Moreover, MS collated the categories into broader patterns of meaning (themes). Discussions among the team resulted in agreement that the overarching theme was around the factors that affect patients’ acceptance of the de-labelling process. After developing and refining the candidate themes, a final set of five themes were agreed by the wider team. The five themes generated provide a story of how patients in the trial experienced PAT, and when and how they accepted the new negative allergy label after PAT.

## Results

### Participants

We invited 40 people, of whom 28 agreed to be interviewed. The interviews were conducted throughout the duration of the trial between May 2022 and December 2023, and lasted between 12 and 51 minutes (average 25 minutes). The median age of participants was 64.5 years (range 24–80 years). We interviewed 20 female participants (71.4%). Out of the 28 participants, 24 had received a negative PAT result and accepted the label; two patients received a positive PAT result; and two patients received a negative PAT result but wished to keep their recorded penicillin allergy status. Of the 24 who had been de-labelled, 10 had been prescribed a penicillin since their PAT.

Five themes capture the patients’ experience of PAT and acceptance of a negative test result and consequent de-labelling and use of penicillin (where relevant).

### Patients identified themselves as being ’low-risk’ before PAT

When patients were asked about their motivations to take part in the trial, most of them reported that they ’*finally*’ wanted to know if they were truly allergic to penicillin. Despite having accepted the allergy status on their medical record, many patients had had long-term doubts about their allergy status but had never had the opportunity to be tested:

*‘Well I was actually fairly confident that I would not be allergic*.’ (P24, 67, female)

The main reason patients doubted their allergy status was their recollection and understanding of the first allergic reaction. Patients had mostly experienced a rash and they acknowledged that they had not experienced anything like an anaphylactic reaction:

*‘I had wondered over the years because I had some sort of infection, so you know, I did wonder sometimes if it might’ve just been a rash that I would’ve had if I wasn’t even taking antibiotics*.’ (P19, 62, female)

Patients reported believing that it wasn’t likely for them to experience a severe allergic reaction during the PAT and most patients did not feel anxious when they attended. Patients thought that if they did have a reaction, it would be similar to the mild reaction they experienced when first labelled as allergic (where patients remembered this event):

*‘I didn’t think it was gonna be a bad* [reaction]*. I knew that it was all controlled, so I wasn’t too worried.’* (P23, 24, female)

### Patients felt trustful and safe during PAT

Patients reported that they felt extremely safe at the hospital. The following three elements contributed to them feeling safe: being informed about the process of PAT and in case of emergency; the belief of how likely they would experience a severe reaction; hospital staff who appeared personable and approachable, and who reassured them during and after PAT.

They reported being well-informed about the procedures involved in testing both at the hospital and when taking penicillin at home, and were happy with information regarding what to do if they experienced any symptoms. This reassurance and information were recognised by the patients to contribute to them being comfortable with and confident in the hospital team and the accuracy of the testing process:

*‘I understood I would be monitored for an appropriate amount of time when exposed to the antibiotic so that I wasn’t sort of left exposed in an unsafe environment*.’ (P28, 31, male)

Patients reported they were well looked after by the hospital staff administering the test. Some patients were also reassured before leaving the hospital that they would very likely receive a negative allergy test result. This verbal reassurance added to the trust in the negative test result letter:

*‘We discussed you know what the outcome might be and of course, as we went along with the different doses and there was no reaction at all then you know it’s probably going to be* [negative] *– it was probably gonna be that I wasn’t allergic and there was a few days as well afterwards where you have to be aware that a reaction can set in*.’ (P24, 67, female)

### Patients’ experiences of any reactions or side effects after PAT

When asked about their views on their negative test result, the majority of patients reported being pleased to know they were not allergic and that they could take penicillin if needed in future. Patients accepted the result as they felt the test was accurate and felt the hospital team was qualified to make the recommendation.

None of the patients that we interviewed who accepted the negative test result reported having any reaction, side effects, or symptoms while undergoing PAT or shortly after.

Two patients experienced symptoms following the test and did not accept the negative test result given to them. Neither patient reported symptoms during the test at the hospital, or when they continued the dose at home. Both patients experienced a mild rash, which started a week after they finished the dose of penicillin at home. On receiving the negative test result letter, the two patients contacted the ALABAMA team about the rash and the negative test result. When they discussed the rash with the research team, including the immunology consultants, they were advised that what they experienced was not a delayed allergic reaction, because of the timeline in which it happened, and that a negative allergy test result was correct.

They both decided to consult their general practice shortly after or at the same time, one spoke to a research nurse and one to their own GP via an e-consult and sent pictures of the rash. In both cases, the research nurse and the GP agreed with the patients that it looked like an allergic reaction and agreed to the decision of not removing the penicillin allergy record. Both patients looked for reliable health and scientific sources online to learn more information about rashes:

*‘I sent photos of it to my GP and he agreed that it looked like an amoxicillin rash, but the team and the immunologist couldn’t possibly think it was because it was a week afterwards.’* (P14, 60, female)

*‘So, he’d* [the consultant] *agreed that I could have further allergy testing if needs be but, obviously, once I’d spoke to the trial nurse at my GP surgery and said, "This is my history," and she said, "Well, I don’t think we need to do any other testing – I think it’s pretty clear that it was the Penicillin!".’* (P10, 38, female)

Patients also compared the rash they experienced after the PAT with the first ‘allergic’ reaction that they could recollect. As the second patient experienced very similar symptoms, this reinforced their interpretation of the rash as an allergic reaction and motivated them to request to keep their penicillin allergy label in their record:

*‘I spoke to the nurse at my GP surgery, and just explained, "I’m really not comfortable with this diagnosis." I said, "I’m aware that I’m not a clinician, but it seems just too weird to me that I would have two identical reactions in my lifetime, both following taking the exact same penicillin-based drug." I was like, "It just doesn’t make any sense to me that it could be anything other than that", and she said, based on my history, it didn’t make any sense to her either.’* (P10, 38, female)

Even when patients understood the difference between allergic reaction and side effect, they did not want to risk experiencing a rash in the future after taking penicillin, and preferred to be cautious and keep the penicillin allergy label and avoid further tests to investigate their reaction further:

*‘I was a little bit cautious to change to non-allergic because I had this nasty rash 10 days later, because if I take penicillin in the future I could have even a worse rash.* […] *The doctor said it didn’t count as an allergy but as a reaction, I may well take it in the future but I could expect a rash, a side effect.’* (P14, 60, female)

### Patients have greater understanding of penicillin allergy reactions versus side effects, during and after PAT

Patients generally reported having a greater understanding of side effects and allergic reactions thanks to the explanations of the hospital testing team and the information leaflet they received as part of the trial. This information had increased their confidence in knowing what to look for when completing the PAT at home and also when taking penicillin in the future:

*‘I would be looking at myself to make sure I was okay and not coming out in a rash or anything*.’ (P28, 31, male)

Patients who accepted the negative result reported that they would be willing to take penicillin antibiotics in the future. Ten out of 24 patients had already taken penicillin at the time of the interview, since being tested. These patients were willing to take penicillin in the future because they understood they would only have a mild side effect, if any at all, based on what they experienced at the hospital and the first time they were labelled as allergic (for example, diarrhoea). The possibility of such a minor side effect was felt acceptable by most as the benefits of taking first-line antibiotics was felt to be worth this. This was especially true for older participants who felt more at risk from serious infections and who prioritised having first-line antibiotics:

*‘I feel good that I can actually take penicillin now. I feel like it may help me to overcome these infections quicker. I feel a lot more confident about the future*.’ (P5, 75, female)

### Patients want clear communication that their allergy record has been changed

Clear communication of the change of their allergy records also supported patients in accepting the de-labelling. Having that conversation with a GP made patients more confident in accepting the new label and in their intention to take penicillin in the future. Patients were reassured when they knew their record had been changed and when a HCP acknowledged and was supportive of this change. Some patients indicated that they would like to discuss the change with their GP, others indicated they would be happy with quick confirmation from their healthcare team; for example, a text message, which one patient was sent by their practice. Some patients just wanted to be reassured that their medical record had been changed and actively followed this up with their practice. A couple of patients checked their online records to see if a change had been made, while others trusted the process and did not need confirmation that their records had been updated:

*‘I’ve been at the surgery just to check that my records are updated. I would have said something, "oh by the way I’ve been part of the ALABAMA study" — and my memory is they would have gone "oh yes" — and I’ve said I wanted to make sure that you know what so that’s me confirming verbally that the record is that I’m okay to have penicillin. I always think face-to-face and voice-to-voice is so much better than paper, even within that non-paper email world*.’ (P20, 79, male)

Some patients wanted reassurance that their record would be updated not only in general practice but also in secondary care and in other health settings such as dentists. In order to facilitate that process patients appreciated having received a card with information about their negative test result and the trial, which they reported carrying around with them ready to use in case they need to discuss their past allergy status outside their practice, such as at the dentist or at the hospital. The card was perceived to facilitate such discussions, especially for older or nervous patients:

*‘To me, it’s important just in case, somewhere if I’m unconscious and somewhere along the line the records haven’t been updated somewhere, then that makes me feel happier that that card will be there to let them know*.’ (P7, 78, male)

## Discussion

### Summary

Our study aimed to investigate patients’ direct experiences of taking part in the ALABAMA PAAP and receiving a negative PAT result. The results of our interviews highlighted the importance of considering the following factors when introducing such programmes:

(1) Our data highlighted how patients make sense of their allergy label and their expectations of how severe a possible allergic reaction during PAT might be. This is in line with previous qualitative research conducted with low-risk penicillin allergy-labelled primary-care patients who had doubts about the accuracy of their label before attending PAT.^15,20^ (2) Our results support the finding that a ’safe clinical environment’ is an important prerequisite for the sustainability and spread of models to deliver services.^21^ (3) Patients need to feel empowered to understand what to look out for when taking penicillin at home, especially in distinguishing between side effects and an allergic reaction. Thanks to that understanding, patients are able to make more informed decisions around the benefits of penicillin antibiotics. (4) Most accepted the possible minor side effects they may experience because they valued the benefits of taking penicillin antibiotics more. (5) Lastly, it is also important to consider effective communication around de-labelling between patients and HCPs in primary care and also with other HCPs such as dentists. The importance of clear communication was also reported in a recent qualitative study on patients’ experiences of de-labelling in hospital, which identified poor information on penicillin allergy and testing as a key barrier to patients undertaking the test.^22^ Effective communication has previously been identified as a pre-requisite of penicillin de-labelling programmes.

### Strengths and limitations

There are several strengths in this study. First, this study is a one of the few evaluations of a trial aimed at amending incorrect penicillin allergy records in primary care. It provides an essential insight into what could affect patients’ acceptance of de-labelling following a negative penicillin allergy test. Second, to ensure rigour in data analysis, several techniques were used, such as having multiple researchers reading transcripts and contributing to the development of categories and themes. We also discussed our coding manual with the researchers from the wider team and refined it. Third, most interviews were conducted by the same interviewer using the same topic guides. This ensured consistency in data collection.

This study also has some limitations. First, it would have been important to understand the views and experiences of patients who are not labelled as ’low-risk’ in order to understand what factors would motivate them to do the test and influence them in accepting a negative label. Another limitation is that we chose to conduct interviews over the telephone to allow us to include patients based across wide geographical regions. While there are limitations to telephone interviews for some types of qualitative studies that might benefit from capturing data on non-verbal communication and/or by establishing a deeper connection via in-person interactions, we did not feel that the remote format significantly influenced the data collection for this study.

### Comparison with existing literature

Our previous work, published elsewhere,^20^ reported the motivations that led patients to undertake PAT, the importance for PAT to be perceived as safe, and patient intentions to take penicillin after a negative PAT result. The current study extends our previous work by including the experience of patients who took penicillin after a negative test and some patients who did not want to be de-labelled. This allowed us to provide a fuller understanding of the factors that are important to consider when introducing penicillin allergy de-labelling programmes.

### Implications for practice

It is essential to understand which factors affect patients’ acceptance of negative PAT results and de-labelling, otherwise algorithms, guidelines, and new allergy pathways may not result in an actual change in allergy status on medical records, as patients may not trust the test result and request to keep their allergy status instead. To enhance that trust, our results highlighted that PAT needs to happen in a safe environment, where patients are also empowered with an understanding of the differences between allergic reactions and side effects. Effective consistent communication about de-labelling between healthcare settings needs to be in place to avoid HCPs contradicting each other.

It is important to consider that effective de-labelling may only work for people who already identify as ’low-risk’ and it may be harder to engage others with potential erroneous records. In this case, we would predict, that patients may be convinced if trusted HCPs tell them they are seen as low-risk. Current systems in primary care are not supporting GPs to identify patients who are low-risk or to discuss penicillin allergy with them. Owing to the lack of allergy testing capacity, de-labelling is only possible with the support of allergy specialists who assess only selected higher-risk patients.

We also recognise that the testing happened in a hospital setting, which was perceived to be ’the safest place to be in case of a severe reaction’*,* and could affect patients’ confidence in the test result itself, meaning that carrying out the PAT elsewhere may lead to more uncertainty among patients. Patients reported feeling safe when they trusted the HCP’s expertise and when they were informed about testing procedures in advance.

Lastly, we recognise the importance of empowering both patients and clinicians with knowledge on the differences between allergic reactions and side effects. GPs in primary care need to be supported in knowing differences between allergic reactions and side effects. This should also be integrated into electronic systems, to easily distinguish between the two, which is not possible in some current electronic systems used in UK general practice.

When challenging erroneous allergy records, it is necessary to create a safe environment for PAT, and to manage patients’ expectations around possible allergic reactions during PAT. If patients identify as low risk, know how to distinguish between allergic reactions and side effects, and are reassured during the test, and have good quality safety information, they are more likely to accept a negative test result. In addition, it is important to confirm with patients that their medical record has been updated, supporting them in communicating the change of allergy status to other care settings outside their general practice.

## Data

The dataset is not publicly available.
